# Efficiency Improvement of Industrial Silicon Solar Cells by the POCl_3_ Diffusion Process

**DOI:** 10.3390/ma16051824

**Published:** 2023-02-23

**Authors:** Xiaodong Xu, Wangping Wu, Qinqin Wang

**Affiliations:** 1 Electrochemistry and Corrosion Laboratory, School of Mechanical Engineering, Changzhou University, Changzhou 213164, China; 2 School of Mechanical Engineering, Yangzhou University, Yangzhou 225127, China

**Keywords:** polycrystalline silicon, solar cells, low-high-low, phosphorus diffusion

## Abstract

To improve the efficiency of polycrystalline silicon solar cells, process optimization is a key technology in the photovoltaic industry. Despite the efficiency of this technique to be reproducible, economic, and simple, it presents a major inconvenience to have a heavily doped region near the surface which induces a high minority carrier recombination. To limit this effect, an optimization of diffused phosphorous profiles is required. A “low-high-low” temperature step of the POCl_3_ diffusion process was developed to improve the efficiency of industrial-type polycrystalline silicon solar cells. The low surface concentration of phosphorus doping of 4.54 × 10^20^ atoms/cm^3^ and junction depth of 0.31 μm at a dopant concentration of *N* = 10^17^ atoms/cm^3^ were obtained. The open-circuit voltage and fill factor of solar cells increased up to 1 mV and 0.30%, compared with the online low-temperature diffusion process, respectively. The efficiency of solar cells and the power of PV cells were increased by 0.1% and 1 W, respectively. This POCl_3_ diffusion process effectively improved the overall efficiency of industrial-type polycrystalline silicon solar cells in this solar field.

## 1. Introduction

Carbon-neutral development strategies have a significant impact on the Earth’s environment, and silicon (Si) solar cells have attracted much attention as a means to use solar energy to convert sunlight into electricity [1,2]. However, a compromise between cost reduction and efficiency improvement must be reached [3]. The PN junction is one of the key technologies in crystalline Si solar cells, which affects photoelectric conversion efficiency. Therefore, the PN junction has excellent performance and stable uniformity. To improve the photoelectric conversion efficiency, the high sheet resistance of over 90 Ω/sq, which has low surface doping concentration and a shallow junction process, was accepted [4]. High open-circuit voltage (*V*_oc_) and short-circuit current (*J*_sc_) values were obtained by this process. Tube furnace diffusion using phosphorus oxychloride (POCl_3_) as a dopant precursor is the dominant emitter formation technology for p-type Si solar cells [5]. The majority of the PV industry currently uses POCl_3_ diffusion to remove metal impurities, including iron [6,7]. The solar cell emitters are obtained by P (phosphorus) diffusion in p-type Si inside of a diffusion tube furnace under controlled conditions of temperature, pressure, and gas flow to form an emitter layer [3].

The photovoltaic (PV) industry has used a quartz diffusion tube furnace to form an emitter layer of the POCl_3_ source. However, there are three flaws in this process [8]: (1) the high sheet resistance, (2) the difficulty in controlling the uniformity of high sheet resistance, and (3) the shallow junction. Many manufacturers tried to reduce the heavy inactive phosphorus concentration and the thickness of the dead zone through an additional step in the industrial process: i.e., chemical etching of the PSG layer after the phosphorus diffusion [9,10,11]. This solution increased the duration of the industrial process and it is expensive. A one-step diffusion process was a common method in which a single temperature and continuous flow of dopant gas were used to deposit phosphor silicate glass (PSG) and to drive dopants to the desired depth [12]. This is a fast process but it tends to create an excessively doped emitter that deteriorates electrical performance [13]. POCl_3_ diffusion could be performed in a two-step process: a PSG deposition step, followed by a drive-in step at variable temperature. During the process, POCl_3_ gas is allowed in the PSG layer, and subsequently, dopants are moved deeply from the PSG layer to the Si substrate in the drive-in step [14]. Wolf et al. [5] presented the status and perspective of emitter formation by the POCl_3_-diffusion process and discussed the diluted source and in-situ post-oxidation technological options for advanced tube furnace POCl_3_-diffusion processes. Cui et al. [15] studied POCl3-based diffusion optimization for the formation of homogeneous emitters and the correlation with metal contact in p-type polycrystalline Si solar cells and found that the sheet resistance is high and that the P surface concentration and emitter saturation current density (*J*_oe_) are low. Cho et al. [16] compared POCl_3_ diffusion and P ion-implantation induced gettering in solar cells and found that the increase in P implantation dose improved the gettering efficiency by increasing bulk lifetime and decreasing iron concentration, but the process remained inferior to POCl_3_ diffusion. POCl_3_-diffused cast quasi-mono cells showed 0.4% higher efficiency due to their higher bulk lifetimes compared to P-implanted emitters. Ghembaza et al. [17] studied the optimization of P emitter formation from POCl_3_ diffusion for p-type Si solar cells and showed that the emitter standard sheet resistances of ~60 Ω/sq and wafer uniformity <3% were obtained from the low-pressure tube furnace. Li et al. [18] investigated POCl_3_ diffusion for the emitter layer formation of industrial Si solar cells and presented the impact of processing parameters on emitter layer formation and electrical performance.

According to the above review, P diffusion can be performed in a single step by controlling a parameter, such as temperature or time [4], or a two-step process, such as ion implantation. In this work, a “low-high-low” (LHL) diffusion process, low-high-low temperature, and three-step diffusion were used to diffuse P elements with different POCl_3_ flows. The ECV profile, open-circuit voltage (*V*_oc_), fill factor (FF), and overall efficiency of solar cells of this process were studied and simultaneously compared with the baseline using the online conventional process.

## 2. Experimental

**Figure 1:**
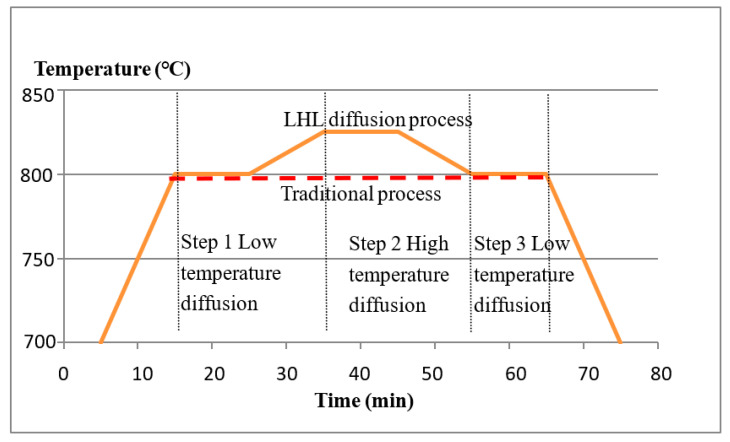
Schematic of BKM and LHL diffusion process.

P diffusion emitters were prepared on 156 × 156 mm 0.5–3 Ω·cm p-type mc-Si wafers with a thickness of ~180 μm in the quartz furnace tube. The distance between the wafers was about 2.35 mm. These wafers were vertically inserted into the quartz boat and then placed in the furnace. There were 1000 pieces per batch. There were two diffusion processes. One was the online process, and another was the LHL diffusion process. Figure 1 shows the schematic of the online and LHL diffusion processes.

Table 1 displays the process parameters of low-temperature online diffusion, namely the BKM (Best Known Method) diffusion process and the LHL diffusion process for solar cells. For the low-temperature online diffusion process, the temperature was kept at 810 °C, and the flow of POCl_3_, O_2,_ and N_2_ gas was 1600 mL/min, 800 mL/min, and 30,000 mL/min, respectively. For the LHL diffusion process, the first step is a low temperature and high POCl_3_ flow diffusion. The low temperature was controlled at about 810 °C, and the flows of POCl_3_, O_2,_ and N_2_ gas were set at 1900 mL/min, 800 mL/min, and 30,000 mL/min, respectively. Next, the high temperature was controlled at 825 °C and the flows of POCl_3_, O_2._ and N_2_ gas were fixed at 2100 mL/min, 800 mL/min, and 30,000 mL/min, respectively. Finally, a low temperature and low POCl_3_ flow diffusion process was used. The diffusion temperature was the same as in the first step. The flows of POCl_3_, O_2,_ and N_2_ gas were 1600 mL/min, 800 mL/min, and 30,000 mL/min, respectively. The three-step variable-temperature diffusion LHL process is useful in the gettering process [19].

Three batches of samples for each process were manufactured in order to get the average values. The sheet resistance was measured using four-point probe equipment, and the P diffusion profiles of selected samples were determined using electrochemical capacitance voltage profiling (ECV-profiling, WEP CVP21). The microstructure and morphology of the textured structure and front metalized areas were observed by scanning electronic microscopy (SEM, Quanta FEG 250, FEI). The electrical properties of solar cells were characterized by a Berger cell tester.

## 3. Results and Discussion

**Figure 2:**
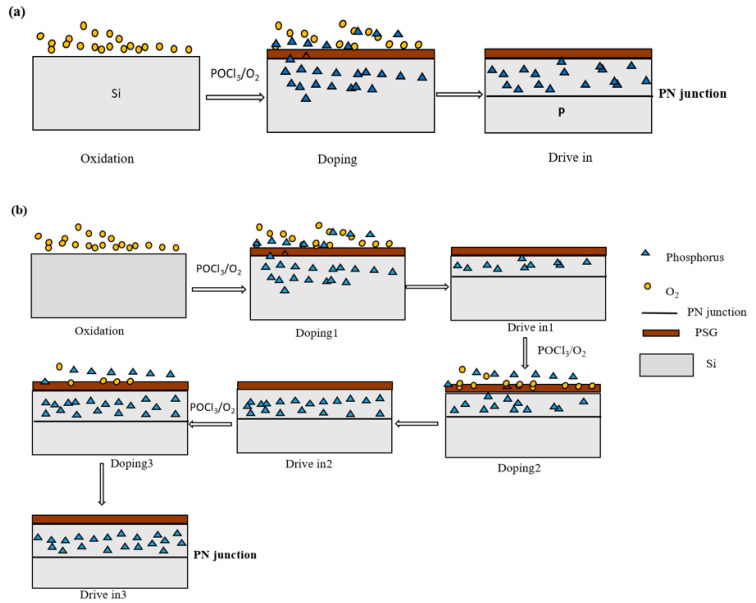
Schematic diagrams of the PN junction of solar cells obtained from (**a**) BKM and (**b**) LHL diffusion processes.

We designed the LHL diffusion process with low-high-low temperature and a three-step diffusion with different POCl_3_ flows. The specific schematic diagrams are shown in Figure 2. Figure 2a shows the schematic diagram of the conventional primary diffusion process. Firstly, pre-oxidation is carried out with oxygen at a low temperature of 700–800 °C to generate silicon oxides on the surface of Si wafers, which is helpful to the distribution of POCl_3_ diffusion. Then, POCl_3_ is deposited at a low temperature of 800 °C, and finally at a high temperature of 850 °C, in order to redistribute the P element. Figure 2b presents the schematic diagram of the LHL diffusion process. LHL diffusion is characterized by three sets of P doping and three sets of redistribution. Variation in temperature is the simplest way to control the phosphorous diffusion profile. As the temperature increases, doping increases, and the formed junctions are deeper. This behavior is explained by the variation of coefficient diffusion and limited solubility with temperature. For this reason, the temperature parameter needed to achieve the necessary exact junction depth has proven to be rather delicate. With a long drive-in time, the junction is deeper. PSG deposited during the pre-deposition step acts as an infinite phosphorus source. All these results confirm that the phosphorus profile is highly affected by the tube furnace conditions. Clearly, time and temperature must be considered carefully. In this work, firstly, the pre-oxidation is carried out with oxygen at a low temperature of 800 °C. Then, the first step of low-temperature POCl_3_ deposition is carried out, and the impurities are redistributed at variable temperatures. Subsequently, the second step of high-temperature POCl_3_ deposition is carried out, which is distributed with high-temperature P impurities. Finally, the third step is to deposit POCl_3_ at a high temperature to cool down, and the impurities are redistributed. LHL diffusion process adopts low-high-low temperature and three-step diffusion with different POCl_3_ flows. In the first step, the low temperature and high POCl_3_ flow are the best to control the tail concentration of ECV curves. In the low-concentration tail, P diffuses into Si wafers primarily via interaction with Si self-interstitials [20,21]. In the second step, the high temperature and high POCl_3_ flow control the kink of the slope. For high P concentration, a conversion from an interstitially to a slow vacancy-mediated process occurs, giving rise to anomalous P diffusion profiles [22]. In the third step, the low temperature and low POCl_3_ flow can control the surface concentration of P doping. This method has the objective to decrease inactive phosphorus through an LHL step. Graphically this implies the reduction of the plateau width, which appears on the top of diffusion profiles near the high-phosphorus concentration zone. The low surface concentration of P doping could be beneficial to the *V*_oc_ and *J*_sc_ values of solar cells. However, it influences series resistance and FF values. Therefore, it is important to weigh the benefits against the risks.

**Figure 3:**
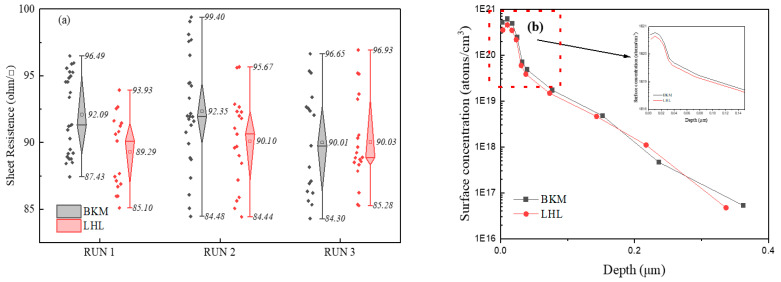
Sheet resistance box-plots of solar cells obtained from BKM and LHL diffusion processes (**a**), and the ECV profiles of P doping (**b**).

Figure 3 shows the sheet resistance box plots of solar cells and the ECV profiles of P doping for solar cells. The sheet resistance of solar cells was obtained (see Figure 3a). It can be observed that solar cells produced from LHL and BKM diffusion processes had the same sheet resistance of about 90 Ω/sq. However, the sheet resistance of solar cells from the LHL process was much more uniform than that of the cells from the BKM diffusion process. The results indicated that the LHL process could be beneficial for the *FF* and the series resistance of solar cells [17]. Figure 3b presents the P doping profile of solar cells produced by LHL and BKM diffusion processes. The solar cells obtained from the LHL diffusion process had a lower surface concentration of P doping, approximately 4.54 × 10^20^ fewer atoms/cm^3^ than those produced from the BKM diffusion process, which produced about 6.08 × 10^20^ atoms/cm^3^ at the junction depth of about 0.02 μm. For LHL and BKM diffusion processes, the solar cells had the same junction depth of around 0.3 μm at a dopant concentration of *N* = 10^17^ atoms/cm^3^. During emitter formation and at high phosphorus concentrations, precipitates were formed on the silicon surface and promoted the existence of electrically inactive phosphorus which formed a dead layer at the silicon surface. This behavior is characterized by a kinked shape in the experimental profiles. This kink has a great impact on solar cell performance since it results in low collection efficiency near the front surface.

**Figure 4:**
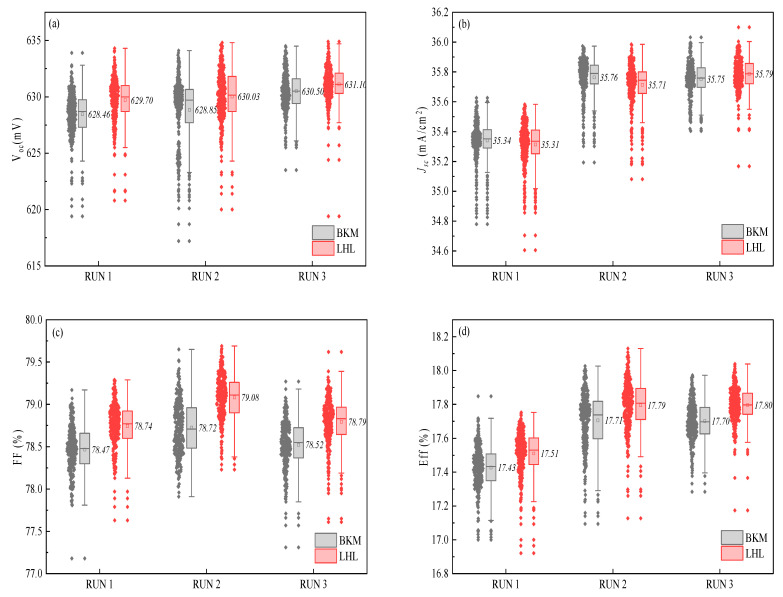
Box plots of the electrical characteristics of solar cells obtained from BKM and LHL diffusion processes (**a**) *V*_oc_, (**b**) *J*_sc_, (**c**) *FF,* and (**d**) *E*_ff_.

In the module of PV solar cell marketing, only the *V*_oc_ and *FF* values have more advantages on the power. In this study, the advantage of the LHL diffusion process could be beneficial to the increase in *V*_oc_ and *FF* values. Table 2 displays the gap of electrical characteristics of solar cells obtained by LHL and BKM diffusion processes. The solar cells obtained from the LHL diffusion process have an increase in median *V*_oc_ value of about 1 mV, compared with the BKM diffusion process. This increase might be due to the low surface concentration of P doping (see Figure 4b). At the same time, the median *FF* value is increased by 0.30%, which can be contributed to the strong impurity absorption effect of Si wafers and the decrease in inactive phosphorus in the LHL diffusion process.

Figure 4 shows the box plots of electrical characteristics of solar cells produced from BKM and LHL diffusion processes. The median *V*_oc_ values of the solar cells produced by LHL and BKM processes are 630 ± 1 mV and 629 ± 1 mV, respectively (see Figure 4a). For LHL and BKM diffusion processes, the median *J_sc_* values of the solar cells are the same, about 35.55 ± 0.25 mA (see Figure 4b). The median FF values of the solar cells obtained by LHL and BKM diffusion processes are 78.9 ± 0.1% and 78.6 ± 0.1%, respectively (see Figure 4c). The median *E_ff_* values of the cells produced by LHL and BKM diffusion processes are 17.65 ± 0.15% and 17.55 ± 0.15%, respectively (Figure 4d). The *E_ff_* value of the solar cells from the LHL diffusion process is increased by 0.08–0.10%, which was mainly attributed to the increase in *V*_oc_ value of 1 mV and the *FF* value of 0.30%. These results show the convergence of the *V*_oc_, *FF* and *E_ff_* values of the solar cells obtained from the LHL diffusion process is better than the parameters of cells produced from the BKM diffusion process, which was attributed to the distribution and diffusion of impurities. At the same time, the effective doping concentration is effectively controlled, the generation of the dead layer is reduced, and recombination is reduced, resulting in the increase of the *V*_oc_ value. Furthermore, due to the LHL diffusion process with three-time temperature changes, it is more conducive to the precipitation of harmful impurities into the PSG layer, which improves the service life of the bulk, thus resulting in the improvement of *FF* values. The increase in Voc and FF can be explained by active phosphorus atoms and the optimized contact-formation process. Table S1 summarizes the performance of solar cells doped P by different diffusion processes (see Supplementary Material).

**Figure 5:**
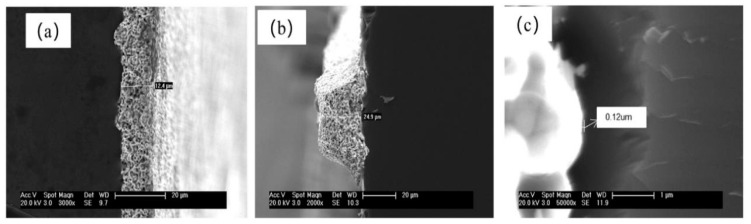
SEM images of (**a**) front busbar, (**b**) front finger, and (**c**) Ag-Si alloy.

**Figure 6:**
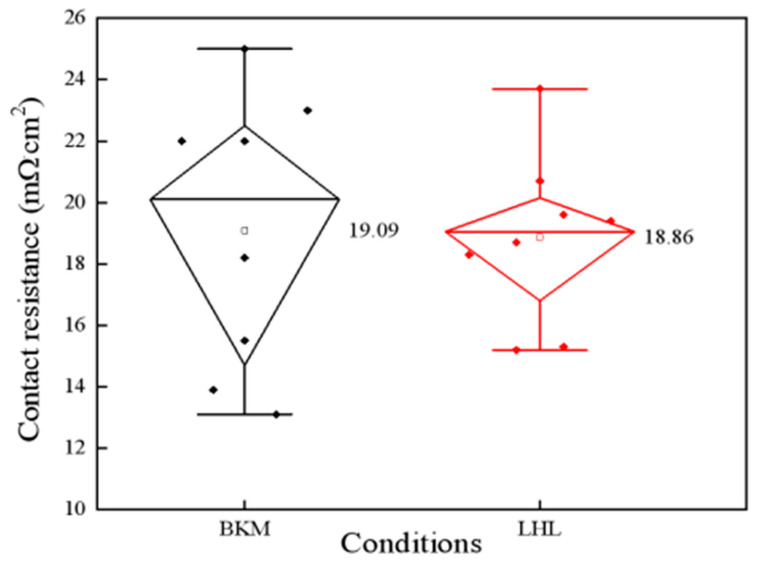
Contact resistivity of solar cells from LHL and BKM diffusion processes.

Figure 5 presents the typical top-view SEM micrographs of the front side of solar cells. It shows that the front busbar is of uniform height: about 12.4 μm (see Figure 5a), and the height of the front finger is also smooth: about 24.3 μm (see Figure 5b). However, the Ag paste does not uniformly corrode the P doping layer. In Figure 5c, the corroded depth is approximately 0.12 μm, so the effect of the surface concentration at the depth of 0.15 μm on the contact resistance (*ρ*_c_) could be emphasized, which is important for the FF values. Figure 6 shows the contact resistivity of solar cells from two diffusion processes. The *ρ*_c_ values of the cells from LHK and BKM processes were 18.86 mΩ∙cm^2^ and 19.09 mΩ∙cm^2^. The LHL diffusion process has no additional costs. It would be a benefit for the large-scale industrial production of the P doping process of PV solar cells.

## 4. Conclusions

The non-uniformity of worm-shaped structures increases the difficulty of P diffusion. In addition, the phosphorus profile is highly affected by the tube furnace conditions. (Time and temperature must be considered carefully). A diffusion process featuring low-high-low temperature and three steps was used to diffuse P elements for solar cells with different POCl_3_ flows in every step. This allows for systematic manipulation of doping profiles, especially for manipulation of the surface-active concentration of P doping, control of the doping depth, and reduction in the dead layer at the silicon surface, respectively. The solar cells with a low surface concentration of P doping of 4.54 × 10^20^ atom/cm^3^ and junction depth of 0.31 μm at a dopant concentration of *N* = 10^17^ atoms/cm^3^ were obtained. The open-circuit voltage and FF values of solar cells increased up to 1 mV and 0.30%, compared with the online low-temperature diffusion process respectively, which can be contributed to the low surface concentration of P doping (decreasing inactive phosphorus) and the strong impurity absorption effect of Si wafers obtained from the low-high-low temperature diffusion process. The efficiency and the power of solar cells were increased by 0.1% and 1 W, respectively. The LHL diffusion process has no additional costs. It would be beneficial for the large-scale industrial production of the P doping process of PV solar cells.

## Figures and Tables

**Table 1:** Process parameters of BKM and LHL diffusion processes for p-type mc-Si solar cells.

Condition		Temperature (°C)	POCl_3_ (sccm)	O_2_ (sccm)	N_2_ (sccm)
**BKM**	1st step	810	1600	800	30,000
**LHL**	1st step	810	1900	800	30,000
	2nd step	825	2100	800	30,000
	Last step	:0	1600	800	30,000

**Table 2:** The gap in electrical characteristics for solar cells obtained by LHL and BKM diffusion processes.

Condition		*V*_oc_ (V)	*J*_sc_ (mA)	*R*_ser_ (ohm)	*FF* (%)	*E*_ff_ (%)
Gap (LHL-BKM)	Run1	0.0012	−0.03	0.0000	0.27%	0.08%
	Run2	0.0012	−0.05	0.0000	0.36%	0.08%
	Run3	0.0006	−0.04	−0.0001	0.27%	0.1%
Average		0.0010	−0.04	0.0000	0.30%	0.09%

Note: *V*_oc_: open circuit voltage; *J*_sc_: short circuit current; *R*_ser_: series resistance; *FF*: fill factor; *E*_ff_: efficiency.

## Data Availability

The data presented in this study are available upon request from the corresponding author.

## Supplementary Materials

The following supporting information can be downloaded at: https://www.mdpi.com/article/10.3390/ma16051824/s1.
