# Supplementary material for: Efficiency Improvement of Industrial Silicon Solar Cells by the POCl3 Diffusion Process
Source: Materials (Basel). 2023 Feb 23;16(5):1824. doi: 10.3390/ma16051824 (PMC10003932; doi:10.3390/ma16051824)
Supplement: Supplementary file 1 [file materials-16-01824-s001.zip › materials-2137846-supplementary.pdf]

Table S1. Summaries of P diffusion process for solar cells from previous publications.

Research contents	Mechanism	Important results	Ref.
Optimize phosphorus-doped emitters	The emitter is the region with the most Auger complexes	The J_{sc} increased from 4.97 to 6.53mA/cm^2, J_{sc} 6.53 mA/cm^2; sheet resistances 60 Ω/sq	[4]
$POCl_3$ diffusion concentration	Reducing the level of surface doping continuously reduces the likelihood of carrier recombination in the emitter.	Carrier recombination was reduced by a factor of three and V_{oc} limit was increased by >30mV, efficiency gain 0.5%; $J_{0,met}$ 200 fA/cm^2; surface concentration of 10^{19} cm^{-3}	[5]
Porous silicon (PS) layer	The heat treatment diffuses P throughout the PS layer and draws metal impurities towards the P-doped PS layer	The mobility and minority carrier diffusion length were significantly enhanced, Temperature 900°C; Time 90 min; Diffusion length 218μm	[6]
Highly doped PSG layer	PSG layer acted as a dopant source during diffusion. The effect of P precipitation at the PSG/Si interface was studied.	The increase in the gas flow of $POCl_3$-N_2 led to a strong accumulation of P at PSG-Si interface. E_{ff} 19.4%; J_{sc} 37.6mA/cm^2; V_{oc} 643mV; FF 80.1%	[8]
P precipitation	The bulk emitter lifetime depends on the presence of P precipitates	The bulk emitter component of the saturation current density depended on the precipitation density, which reduced the open circuit voltage of the cell, Emitter lifetime 10^{-11} s	[12]
P precipitate	Precipitation leads to P electricity inactivation in Si	High density of small coherent precipitation; Precipitation was associated with a very high enhancement of P diffusivity	[13]
Elimination of emitter inactive P	SRH recombination caused by inactive P atoms is a major limitation.	Free P oxidation was the main mechanism for inhibiting inactive P, regulating O_2 flow rate, and controlling the surface P concentration, J_{sc} 51 fA/cm^2	[14]
$POCl_3$ diffusion optimized emitter	Highly doped selective electrodes, combined with thin and high metal electrodes, make it easier to collect carriers.	Higher V_{oc} and $J_{sc,}$ lower fill factor (FF). optimal R_{sheet} 90 ohm/square; E_{ff} 18.325%	[15]
P ion implantation	The formation of P emitters can absorb impurities such as Fe.	Higher body life; Lower $J_{sc,}$ ~0.3% higher absolute efficiency.	[16]
$POCl_3$ diffusion	$POCl_3$ offers high throughput, impurity absorption and reliability	Provided a feasible solution to solve the emitter SRH composite and surface SRH compounding, doping concentration of 2×10^{20} cm^{-3}; diffusion temperature 830°C.	[18]
$POCl_3$ diffusion	Highly doped regions result in high minority carrier recombination	The diffusion time and temperature control the junction depth and the mass of the emitter formed. Performance improvement 2.78%; E_{ff} 16.5%	[19]
$POCl_3$ diffusion "LHL" temperature step	Manipulate the surface doped P concentration and doping depth	The V_{oc}, FF, and efficiency increased by 1 mV, 0.30%, and 1%; dopant concentration 10^{17} atoms/cm^3	This work
